# Adenovirus-Associated Influenza-Like Illness among College Students, Pennsylvania, USA

**DOI:** 10.3201/eid2411.180488

**Published:** 2018-11

**Authors:** Holly M. Biggs, Xiaoyan Lu, Lisa Dettinger, Senthilkumar Sakthivel, John T. Watson, Sameh W. Boktor

**Affiliations:** Centers for Disease Control and Prevention, Atlanta, Georgia, USA (H.M. Biggs, X. Lu, J.T. Watson);; Pennsylvania Department of Health, Harrisburg, Pennsylvania, USA (L. Dettinger, S. Boktor);; Batelle, Atlanta (S. Sakthivel)

**Keywords:** human adenoviruses, human adenovirus infections, adenovirus vaccines, public health surveillance, epidemiology, student health services, viruses, Pennsylvania, viruses, respiratory infections, influenza-like illness, students, United States

## Abstract

Among students with influenza-like illness at a Pennsylvania college student health center during 2016–2017, 44 (15%) of 288 with respiratory specimens tested positive for human adenovirus (HAdV). HAdV-3, -7, and -4 predominated, and types clustered temporally. HAdV infection should be considered among college students with acute respiratory illness.

Human adenoviruses (HAdVs) cause a range of clinical manifestations, most commonly acute respiratory illness (ARI), gastroenteritis, and conjunctivitis. Seven HAdV species (A–G) and >80 types are known to cause human infection, and certain HAdV types are associated with particular tissue tropisms and clinical syndromes (*1*). Outbreaks of HAdV infection occur in a variety of settings, including schools, long-term care facilities, military recruit training facilities, and the civilian community (*2*–*4*). The substantial impact of HAdV ARI among US military recruits drove development of the first live oral vaccine for HAdV types 4 and 7 for military use. After vaccine introduction in 1971, and again after reintroduction in 2011, dramatic declines were documented in respiratory illness among recruits (*5*). Currently, the HAdV vaccine for types 4 and 7 is licensed in the United States for use in military personnel 17–50 years of age and is administered routinely at all US basic military training sites (*5*). However, despite some similarities between military recruits and civilian college students, including age and sharing of residences, little is known about the contribution of HAdV to respiratory illness in college students.

The Pennsylvania Department of Health (PDH) conducts surveillance for influenza-like illness (ILI), defined as fever (temperature >100°F [>37.8°C]) plus cough or sore throat without a known cause other than influenza, at participating outpatient healthcare facilities throughout the state. Basic demographic information is recorded, and from a convenience sample of cases, a nasopharyngeal swab specimen is collected. These specimens are tested by the PDH Bureau of Laboratories using Centers for Disease Control and Prevention (CDC) real-time reverse transcription PCR for influenza A and B, HAdV, respiratory syncytial virus, human metapneumovirus, rhinovirus, and parainfluenza virus types 1–3.

We describe HAdV types associated with ILI among students who sought care at a student health center (SHC) on a large college campus during August 28, 2016–August 26, 2017. Specimens identified as HAdV-positive among students with ILI at the SHC were sent to CDC to determine HAdV species and type. Molecular typing was performed by PCR and sequencing of the hexon hypervariable regions 1–6 (*6*).

During the study period, 1,149 ILI cases were reported from the SHC; for 288 (25%), a nasopharyngeal swab specimen was tested for respiratory viruses (Figure, panel A). Of these, 44 (15%) specimens were positive for HAdV. Three HAdV species and 4 HAdV types were detected: HAdV-3 and HAdV-7 of species HAdV-B in 21 (48%) and 16 (36%), respectively; HAdV-4 of species HAdV-E in 5 (11%); and HAdV-1 of species HAdV-C in 2 (5%). The median age of HAdV-positive students was 19 years (range 18–27 years), and 31 (70%) were male. Among HAdV-positive specimens, rhinovirus was co-detected in 4 and parainfluenza virus type 2 in 1. 

**Figure:**
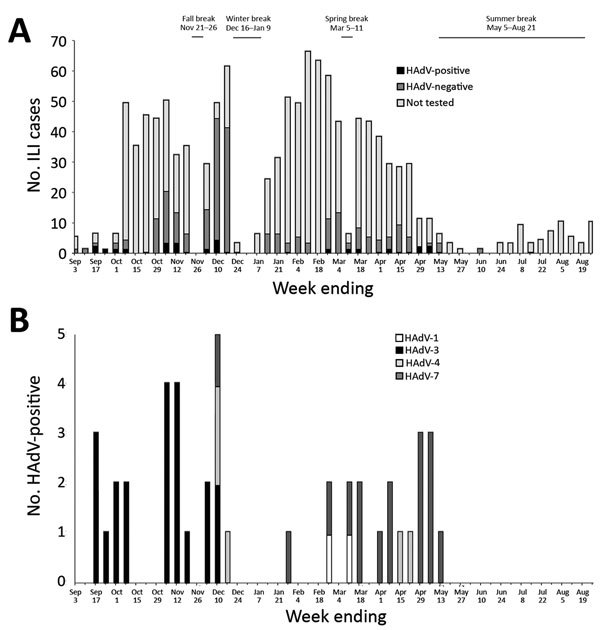
Adenovirus-associated ILI among students at a state college, Pennsylvania, USA, August 28, 2016–August 26, 2017. A) ILI cases from the student health center (SHC) and weekly number of human adenovirus (HAdV)–positive, –negative, and not tested cases. B) HAdV types identified from the SHC. HAdV detections were aggregated by epidemiologic week based on specimen collection date. Fall semester 2016 began August 22, 2016; spring semester 2017 ended May 5, 2017; fall semester 2017 began August 21, 2017. ILI, influenza-like illness.

HAdV-3 was identified during September–December; no additional HAdV-3 was identified after the 4-week winter break. HAdV-7 and -4 were first detected in December, before winter break, then throughout the spring; HAdV-7 was the most commonly detected type during this period (Figure, panel B).

The HAdV types detected from the SHC are recognized causes of ARI. HAdV-3 was the most commonly reported HAdV type in the United States during 2003–2013 (*7*). HAdV-4 and -7 are increasingly recognized as causes of respiratory illness in the community and other nonmilitary populations (*7*–*9*); HAdV-7, in particular, has been associated with severe respiratory illness in adults. A male predominance was observed among HAdV-positive students at the SHC; this finding has been previously described, but its significance is unknown (*2*,*4*). 

HAdVs circulate throughout the year without a discernable seasonality, although some reports describe a winter and spring predominance of respiratory outbreaks (*1*). The temporal clustering of types that we observed might reflect transmission dynamics within the college population rather than viral seasonal patterns per se; however, additional surveillance may help further define circulation patterns of HAdV types (*8*). In a comparable report from New York, during the 2014–15 influenza season, ≈8% of samples tested from students with ILI at 1 college were positive for HAdV; HAdV-14 and -4 were most frequently detected (*10*).

Our findings are subject to several limitations. Specimens tested for respiratory viruses represent a convenience sample of ILI cases, and the proportion sampled varied by week. We report findings from surveillance at a single college SHC; phylogenetic analysis of hexon sequences was not conducted, preventing comparisons between the detected virus strains and those reported to circulate in military or other civilian communities.

Although recruits at basic military training are recognized to be at risk for infection with HAdV, less is known about the risk for HAdV in nonmilitary congregate settings. We detected HAdV in a substantial proportion of ILI cases among a convenience sample of young adults at an SHC surveillance site at a large university during the 2016–17 academic year. Understanding the effects of HAdV respiratory illness on college campuses, including severity, missed class time, and occurrence of outbreaks, would be useful in assessing potential control measures in these settings.
